# Genome-wide survey indicates diverse physiological roles of the turnip (*Brassica rapa* var. *rapa*) calcium-dependent protein kinase genes

**DOI:** 10.1038/s41598-017-16102-0

**Published:** 2017-11-17

**Authors:** Qiuli Wang, Xin Yin, Qian Chen, Nan Xiang, Xudong Sun, Yunqiang Yang, Yongping Yang

**Affiliations:** 1 Key Laboratory for Plant Diversity and Biogeography of East Asia, Kunming Institute of Botany, Chinese Academy of Science, Kunming, 650204 China; 2 Plant Germplasm and Genomics Center, Kunming Institute of Botany, Chinese Academy of Sciences, Kunming, 650201 China; 3 Institute of Tibetan Plateau Research at Kunming, Kunming Institute of Botany, Chinese Academy of Sciences, Kunming, 650201 China; 4 School of Life Sciences, Yunnan University, Kunming, 650091 China; 5 University of Chinese Academy of Sciences, Beijing, 100049 China

## Abstract

Calcium-dependent protein kinases (CDPKs) as crucial sensors of calcium concentration changes play important roles in responding to abiotic and biotic stresses. In this study, 55 *BrrCDPK* genes, which were phylogenetically clustered into four subfamilies, were identified. Chromosome locations indicated that the CDPK family in turnip expanded by segmental duplication and genome rearrangement. Moreover, gene expression profiles showed that different *BrrCDPKs* were expressed in specific tissues or stages. Transcript levels of *BrrCDPKs* indicated that they were involved in abiotic and biotic stresses and that paralogs exhibited functional divergence. Additionally, we identified 15 *Rboh* genes in turnip; the results of yeast two-hybrid analysis suggested that BrrRbohD1 interacted only with BrrCDPK10 and that BrrRbohD2 interacted with BrrCDPK4/7/9/10/17/22/23. Most of the genes play an important role in *pst* DC3000 defense by regulating the accumulation of H_2_O_2_ and stomatal closure. Our study may provide an important foundation for future functional analysis of BrrCDPKs and reveal further biological roles.

## Introduction

Calcium, a universal second messenger, plays an important role in the signal transduction pathways that were developed to survive developmental and stress stimuli in eukaryotes^1,2^. In plants, Ca^2+^ changes are sensed and decoded by Ca^2+^ sensors or Ca^2+^-binding proteins that transduce signatures into a series of downstream effects^3,4^. Four classes of sophisticated Ca^2+^ sensors or Ca^2+^-binding proteins exist, including calmodulins (CaM), calmodulin-like proteins, calcineurin B-like proteins, and calcium-dependent protein kinases (CDPK)^5–8^. CDPKs are unique in having both protein kinase and calmodulin-like domains in a single polypeptide that directly result in Ca^2+^-binding and Ca^2+^-stimulated kinase activities without independent CaM^9,10^.

CDPKs forms large multigene families; 34, 31, 26, 35, 25, and 50 CDPKs have been identified in *Arabidopsis thaliana* ^11^, rice^12^, wheat^13^, maize^14^, canola^15^, and soybean^16^, respectively. A typical CDPK protein contains a variable N-terminal domain, a catalytic Ser/Thr protein kinase domain, an autoinhibitory domain, and a CaM domain^5,11^. The variable N-terminal domain may be important for substrate recognition^17^ and usually has palmitoylation or myristoylation sites that are related to membrane localization^9^. The protein kinase domain is the catalytic domain containing an adenosine triphosphate binding site and lies adjacent to the autoinhibitory junction domain. The consecutive calmodulin-like domain always contains four EF hands for Ca^2+^ binding and is the reason why CDPKs rely on Ca^2+^ and not calmodulin^11^.

Many studies have reported that CDPKs are involved in plant responses to biotic and abiotic stresses and signal the transduction of hormones^11,12,18,19^. In *Arabidopsis*, *AtCDPK1* and *AtCDPK2* mRNA expression were rapidly induced by drought and high salt^20^. AtCDPK4/5/6/11 regulated microbe-associated molecular patterns (MAMPs) or pathogen-associated molecular patterns that triggered immunity and phosphorylated WRKY transcription factors to regulate gene expression^21^. Moreover, by working together with their substrates^22^, CDPKs have diverse functions in carbon and nitrogen metabolism, phospholipid synthesis, defense responses, ion and water transport, cytoskeleton organization, transcription, and hormone responses^23^. Many transcription factors, such as abscisic acid (ABA)-responsive element-binding factor (ABF), repression of shoot growth (RSG), and heat shock factor B2a, are confirmed substrates of CDPK. AtDi19-2, a drought-induced protein family member, was strongly phosphorylated by AtCPK16^24^. AtCPK32 regulated ABA-responsive gene expression via ABF4^25^. NtCDPK1 interacted with NtRSG and phosphorylated Ser-114 of NtRSG^26^. Additionally, Rboh was a substrate of CDPK^27^. In *Arabidopsis*, AtCDPK1/2/4/11 phosphorylated Rbohs to induce the production of reactive oxygen species (ROS)^28^ that played a positive role in resistance to various pathogens by regulating the initiation of programmed cell death or by promoting the salicylic acid (SA) signaling pathway^28,29^. Furthermore, NtCDPK2/3 and StCDPK4/5 were biochemically activated and participated in the induction of early defense responses by phosphorylating RbohB after exposure to biotic stress^30,31^.

Turnip (*Brassica rapa* var. *rapa*) is a subspecies of *B. rapa* ^32^ and an important traditional crop plant in the Tibet Plateau. The tuberous root of turnip is used as food, forage, and medicine. It has been used for human consumption worldwide^33^. Turnip can also grow well in cold regions^34^. *CDPK* genes are promising candidates for modification of plant stress tolerance. However, few studies on turnip are available. Thus, we initiated a study to isolate *CDPKs* from turnip. In this study, we determined the number of *CDPK* genes in turnip and analyzed phylogeny, chromosome locations, divergence time, expression profiles, and transcriptional levels. Furthermore, we studied the relationship between BrrCDPKs and BrrRbohs using leaf inoculation with *pst* DC3000. Our results provide important information on the evolutionary history and the biological functions of the turnip CDPK family.

## Results

### Identification of the *CDPK* gene family in turnip

To find all *CDPKs* in turnip, a genome-wide analysis of the *CDPK* gene family was performed with the assistance of the Turnip Genome Database in the JBrowse website. A total of 72 proteins with a protein kinase domain and at least one EF-hand domain were identified. Up to 17 of them that were similar to CDPK-related proteins, calcium/calmodulin-dependent proteins, and calcium and calcium/calmodulin-dependent protein kinases were eliminated; the remaining proteins were considered to be BrrCDPKs. We then named the remaining genes *BrrCDPK1* to *BrrCDPK55* according to the position of the genes on chromosomes (Table 1). Additionally, we obtained detailed gene information. Of these 55 genes, 52 *BrrCDPK* genes contained four EF-hand domains, with *BrrCDPK5*, *BrrCDPK25*, and *BrrCDPK45* having only three, one, and three EF-hands, respectively. The BrrCDPKs identified in our study ranged in molecular mass from 38.45 kDa to 106.2 kDa, and all contained the typical CDPK structure. Furthermore, 38 CDPKs contained myristoylation sites and 47 of the 55 BrrCDPK proteins possessed palmitoylation sites at their N-termini (Table 1). CDPK proteins possessing myristoylation and palmitoylation motifs are related to their subcellular localization and substrate specificity^5,35^.

**Table 1:** *CDPK* gene family in turnip.

Gene	Gene Locus	CDS (bp)	No. of EF hands	MW (KDa)	pI	GRAVY	N-Myr	N-Pal	N-Term
BrrCDPK1	chr1:1427121..1430241(−)	1638	4	61.16	5.48	−0.347	Y	Y	N
BrrCDPK2	chr1:6395129..6397523(+)	1722	4	64.88	5.71	−0.525	Y	Y	N
BrrCDPK3	chr1:7348109..7350449(−)	1578	4	58.8	6.09	−0.541	Y	N	N
BrrCDPK4	chr1:8994725..8997312(−)	1461	4	54.99	5.29	−0.298	N	Y	N
BrrCDPK5	chr1:22510976..22514438(+)	1581	3	59.79	5.28	−0.452	N	Y	N
BrrCDPK6	chr2:3018873..3022140(+)	1542	4	57.66	5.69	−0.416	N	N	Y
BrrCDPK7	chr2:4702301..4706984(−)	1746	4	57.73	5.56	−0.487	Y	Y	N
BrrCDPK8	chr2:4737776..4740369(−)	1605	4	60.14	5.99	−0.445	Y	Y	N
BrrCDPK9	chr2:25412374..25416270(+)	1467	4	55.34	5.25	−0.357	N	Y	N
BrrCDPK10	chr2:25416270..25420163(+)	1281	4	48.02	5.37	−0.26	N	Y	N
BrrCDPK11	chr2:25420163..25422289(+)	1467	4	55.41	5.17	−0.42	N	Y	N
BrrCDPK12	chr3:795109..797500(−)	1800	4	66.74	5.23	−0.398	Y	Y	N
BrrCDPK13	chr3:2194413..2196401(+)	1572	4	58.23	5.66	−0.486	Y	Y	N
BrrCDPK14	chr3:7345714..7348731(+)	1746	4	66.04	6.36	−0.437	Y	Y	N
BrrCDPK15	chr3:9231301..9233800(−)	1701	4	62.86	5.45	−0.385	Y	Y	N
BrrCDPK16	chr3:12406317..12408965(+)	1503	4	56.28	5.21	−0.362	N	N	N
BrrCDPK17	chr3:15992546..15997951(−)	2880	4	106.2	5.67	−0.318	Y	N	N
BrrCDPK18	chr3:18412320..18414842(+)	1629	4	60.71	5.64	−0.513	Y	Y	N
BrrCDPK19	chr3:21931910..21934109(+)	1587	4	59.45	6.2	−0.413	Y	Y	N
BrrCDPK20	chr3:25161436..25179997(−)	1599	4	59.48	6.31	−0.52	Y	N	N
BrrCDPK21	chr3:29788001..29794408(+)	1647	4	61.58	5.68	−0.344	Y	Y	N
BrrCDPK22	chr3:30065232..30067756(−)	1626	4	61.54	7.2	−0.584	Y	Y	N
BrrCDPK23	chr4:4423201..4426117(−)	1587	4	59.51	6.67	−0.416	Y	Y	N
BrrCDPK24	chr4:14104630..14111261(−)	1770	4	67.48	5.86	−0.421	N	Y	N
BrrCDPK25	chr4:15518281..15520338(−)	1548	1	58.28	5.22	−0.605	N	Y	N
BrrCDPK26	chr4:17611402..17613847(−)	1596	4	60.16	7.62	−0.413	Y	Y	N
BrrCDPK27	chr5:1238237..1240655(−)	1599	4	60.27	6.57	−0.381	Y	Y	N
BrrCDPK28	chr5:6815494..6817962(−)	1755	4	66.3	6.41	−0.482	Y	Y	N
BrrCDPK29	chr5:6846637..6848824(+)	1290	4	48.25	5.64	−0.465	Y	Y	N
BrrCDPK30	chr5:8688703..8691245(−)	1563	4	58.65	6.16	−0.463	Y	Y	N
BrrCDPK31	chr5:13486470..13488611(−)	1494	4	56.13	5.22	−0.355	N	Y	N
BrrCDPK32	chr5:21874565..21880352(+)	2841	4	104.74	5.54	−0.349	N	Y	N
BrrCDPK33	chr6:7326856..7329517(−)	1638	4	61.31	6.08	−0.333	Y	Y	N
BrrCDPK34	chr6:16110279..16113027(+)	1746	4	65.7	9.34	−0.65	Y	Y	N
BrrCDPK35	chr6:16346677..16349111(−)	1620	4	60.57	5.19	−0.373	Y	Y	N
BrrCDPK36	chr6:16558944..16564439(−)	2763	4	103.93	6.53	−0.497	N	Y	N
BrrCDPK37	chr6:26153739..26159782(+)	2688	4	101.89	6.38	−0.289	N	Y	N
BrrCDPK38	chr7:10206195..10208957(+)	1584	4	59.61	8.75	−0.489	N	N	N
BrrCDPK39	chr7:12333863..12336698(−)	1617	4	60.84	5.88	−0.534	Y	Y	N
BrrCDPK40	chr7:20243361..20245669(−)	1641	4	61.94	6.35	−0.411	Y	Y	N
BrrCDPK41	chr7:20745194..20750493(+)	1584	4	59.51	5.76	−0.473	Y	Y	N
BrrCDPK42	chr9:4100343..4103209(−)	1617	4	60.81	8.95	−0.531	Y	N	N
BrrCDPK43	chr9:4562547..4566596(+)	1653	4	61.64	5.22	−0.363	Y	N	N
BrrCDPK44	chr9:17398820..17401327(+)	1446	4	54.34	5.48	−0.364	Y	Y	N
BrrCDPK45	chr9:17401578..17404101(+)	1038	3	38.45	5.42	−0.427	N	Y	N
BrrCDPK46	chr9:25902015..25904900(+)	1587	4	59.43	6.5	−0.405	Y	Y	N
BrrCDPK47	chr9:28600885..28603862(−)	1626	4	61.34	6.25	−0.475	Y	Y	N
BrrCDPK48	chr9:32643372..32645805(+)	1596	4	60.15	6.63	−0.378	Y	Y	N
BrrCDPK49	chr10:10265097..10267598(−)	1485	4	55.75	5.04	−0.373	N	Y	N
BrrCDPK50	chr10:10687217..10690252(+)	1437	4	54.17	5.6	−0.402	N	Y	Y
BrrCDPK51	chr10:10715953..10718319(+)	1554	4	57.67	5.45	−0.451	Y	Y	N
BrrCDPK52	chr10:11422385..11427765(+)	2676	4	96.97	8.81	−0.565	Y	Y	N
BrrCDPK53	chr10:14191675..14201474(+)	1599	4	59.46	5.92	−0.418	Y	Y	N
BrrCDPK54	chr10:14320716..14323063(−)	1569	4	57.84	5.82	−0.444	Y	Y	N
BrrCDPK55	chr10:16497118..16499567(+)	1776	4	65.76	5.38	−0.394	Y	Y	N

GRAVY, grand average of hydropathicity; **N-Myr**, myristoylation site; **N-Pal**, palmitoylation site; **N-Term**, N-terminal acylation; N, NO; Y, Yes.

### Phylogenetic relationships and exon–intron structure analysis

**Figure 1:**
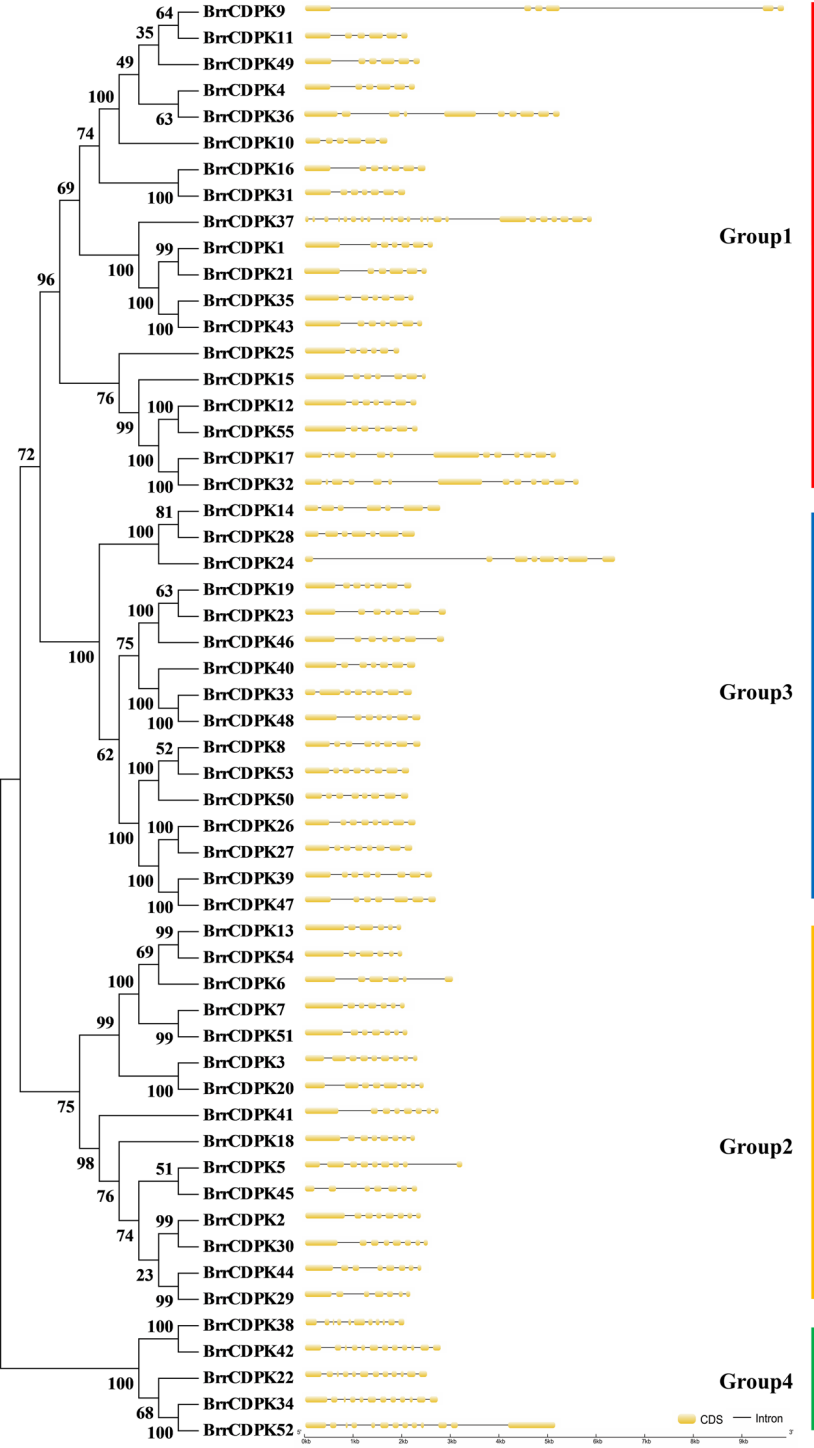
Phylogenetic relationship and gene structure of turnip CDPKs. A neighbor-joining tree was created for 55 turnip CDPK proteins using the MEGA7.0 program with 1000 bootstrap replicates. Four subfamilies were labeled Groups 1–4 with vertical bars in specific colors. Exons and introns are represented by yellow boxes and black lines, respectively.

To investigate the phylogenetic relationship of *CDPK* genes, a neighbor-joining tree was constructed by CDPK protein sequences from turnip (Fig. 1). According to the phylogenetic tree, all CDPKs from turnip were divided into four major subfamilies based on the tree topology (Groups 1, 2, 3, and 4); this agreed with the classification of *Arabidopsis CDPKs* ^11^. As exon/intron structures and the types and numbers of introns can demonstrate the evolutionary history of some gene families^36^, the exon/intron structure of all 55 *BrrCDPKs* was analyzed to gain further insight. As Fig. 1 shows, most of the members of Group 1 had five to six introns except BrrCDPK17, BrrCDPK32, BrrCDPK36, and BrrCDPK37, whereas those in Group 3 contained six to seven introns. Additionally, five to seven and 10 to 12 introns were found in Groups 2 and 4, respectively. In general, BrrCDPKs that clustered in the same subfamily showed similar exon/intron structures, indicating their close evolutionary relationships and suggesting that gene family expansion occurred via ancient paralogs or multiple origins of gene ancestry.

### Chromosomal distributions of *BrrCDPKs*

**Figure 2:**
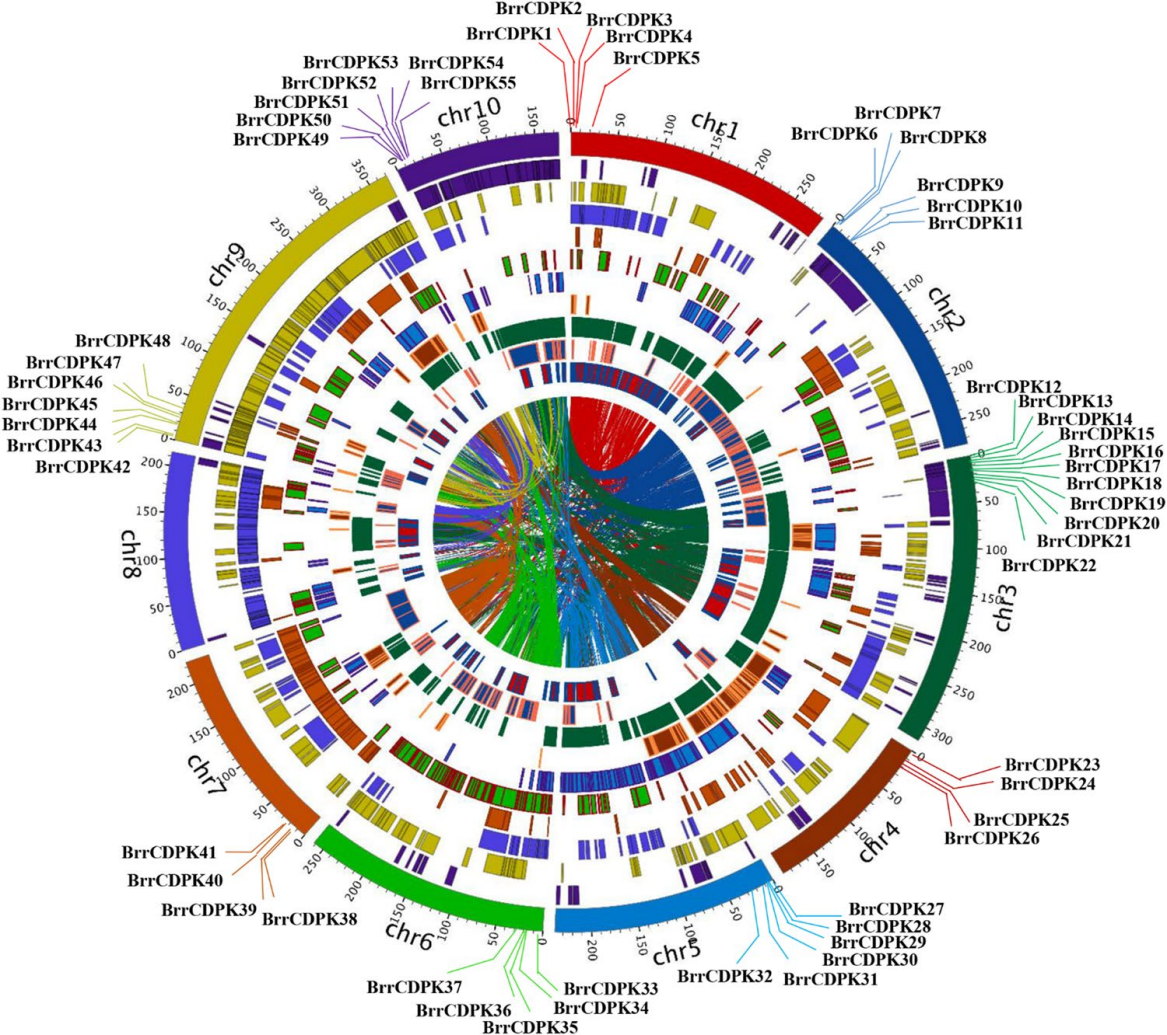
*BrrCDPKs*’ chromosome distributions, synteny blocks, and the turnip genome duplication event caused paralogous relationships. Chromosomes are shown in different colors and in the outer circle, where the numbers represent the chromosome length in 100 Kb. The *BrrCDPK* genes are marked at their approximate positions with specific colored lines on the circle. Filled blocks in different colors denote the syntenic relationships of turnip *CDPK* genes.

To understand the genomic distribution of the predicted *BrrCDPKs*, the DNA sequences were used to search the turnip genome database. As seen in Fig. 2, chromosome 8 was the only one of the 10 chromosomes that carried no *BrrCDPK* genes, while only chromosome 3 had 11 *BrrCDPK* genes, the maximum number found on one chromosome. Meanwhile, chromosomes 4 and 7 contained four *BrrCDPKs*, chromosomes 1 and 6 carried five *BrrCDPKs*, chromosomes 2 and 5 contained six *BrrCDPK* genes, and chromosomes 9 and 10 had seven *BrrCDPK* genes. Interestingly, all *BrrCDPKs* were located in front of the chromosomes and most adjacent genes were arranged densely. Moreover, a large number of segmental duplications were found between genes and chromosomes. For instance, *BrrCDPK12* was close to *BrrCDPK13* on chromosome 3 and *BrrCDPK54* was next to *BrrCDPK55* on chromosome 10. Furthermore, *BrrCDPK12* and *BrrCDPK55* were paralogs, as were *BrrCDPK13* and *BrrCDPK54*.

### Evolutionary history of the turnip *CDPK* gene family

To estimate the divergence time of turnip *CDPKs*, synonymous (*Ks*) and nonsynonymous (*Ka*) mutation rates were measured between predicted orthologous *CDPK* genes. We used a divergence rate of 1.5 × 10^−8^ mutations per *Ks* site per year^37^ to estimate the divergence time of 21 pairs of *CDPK* orthologs. We found that the estimated time was between 5.740 and 28.343 MYA (Table 2). The divergence time of six *BrrCDPK* orthologous genes (*BrrCDPK2* and *BrrCDPK30*; *BrrCDPK8* and *BrrCDPK53*; and *BrrCDPK16* and *BrrCDPK31*) was not included in the next calculation because the estimated separation time of these genes (28.35, 28.3433, and 27.0867 MYA) was much earlier than the known speciation time for *Brassica* and *Arabidopsis* (14.5–20.4 MYA)^38^. The orthologs of *BrrCDPK5* and *BrrCDPK45* had no *Ks* value, indicating that they did not show synonymous substitution. Thus, we cannot estimate divergence time. The average time of the remaining orthologs was calculated at ~11 MYA with a standard deviation of 3 MYA. Additionally, the *Ka/Ks* (ω) value of all the predicted *CDPK* paralogs that had average values of 0.076 was less than one, suggesting that the 20 pairs of CDPK proteins were under strong purifying selection pressure.

**Table 2:** Estimated divergence time between turnip *CDPK* orthologs.

Seq. 1	Seq. 2	Identity (%)	*Ks*	*Ka*	ω	T(MYA)
BrrCDPK1	BrrCDPK21	89.07	0.4562	0.0179	0.0392	15.2067
BrrCDPK2	BrrCDPK30	75.39	0.8505	0.0444	0.0522	28.3500
BrrCDPK3	BrrCDPK20	92.29	0.4073	0.0025	0.0061	13.5767
BrrCDPK4	BrrCDPK36	46.85	0.5267	0.0431	0.0818	17.5567
BrrCDPK7	BrrCDPK51	94.43	0.3429	0.0051	0.0149	11.4300
BrrCDPK8	BrrCDPK53	88.62	0.8503	0.0459	0.0540	28.3433
BrrCDPK9	BrrCDPK11	90.78	0.1722	0.0338	0.1963	5.7400
BrrCDPK12	BrrCDPK55	91.69	0.4279	0.0127	0.0297	14.2633
BrrCDPK13	BrrCDPK54	93.88	0.2903	0.0141	0.0486	9.6767
BrrCDPK14	BrrCDPK28	88.87	0.2955	0.0483	0.1635	9.8500
BrrCDPK16	BrrCDPK31	93.6	0.8126	0.0102	0.0126	27.0867
BrrCDPK17	BrrCDPK32	87.95	0.2939	0.0051	0.0174	9.7967
BrrCDPK19	BrrCDPK23	97.16	0.3988	0.0102	0.0256	13.2933
BrrCDPK26	BrrCDPK27	90.04	0.3548	0.0284	0.0800	11.8267
BrrCDPK29	BrrCDPK44	53.27	0.5418	0.2349	0.4336	18.0600
BrrCDPK33	BrrCDPK48	89.8	0.3311	0.0179	0.0541	11.0367
BrrCDPK34	BrrCDPK52	58.14	0.228	0.0282	0.1237	7.6000
BrrCDPK35	BrrCDPK43	93.45	0.2464	0.0076	0.0308	8.2133
BrrCDPK38	BrrCDPK42	92.57	0.1915	0.0051	0.0266	6.3833
BrrCDPK39	BrrCDPK47	91.33	0.2968	0.0102	0.0344	9.8933

*Ks*: synonymous substitution rate; *Ka*: nonsynonymous substitution rate; ω: *Ka/Ks*; MYA: million years ago.

### Development-related expression profiles of *BrrCDPK* genes

**Figure 3:**
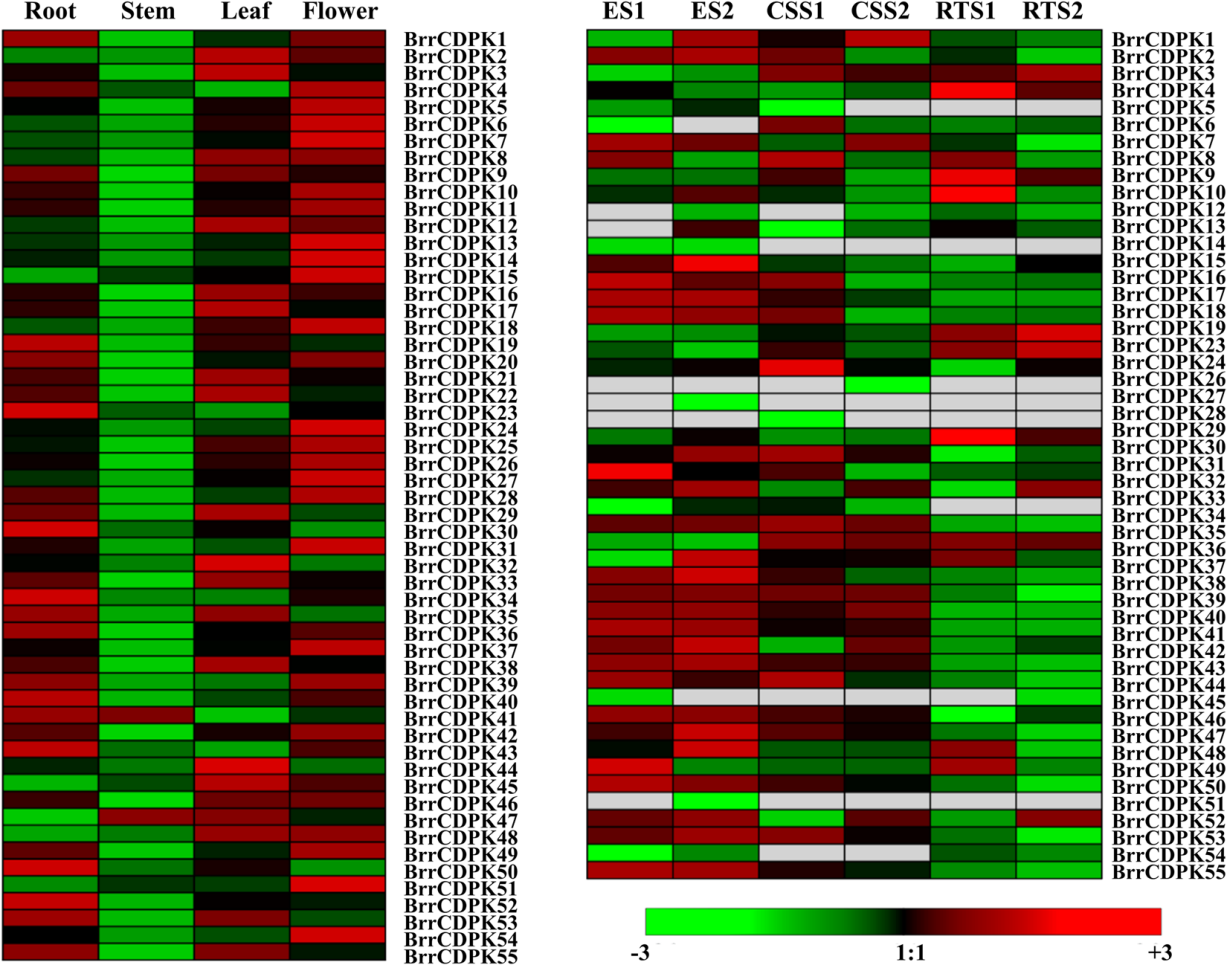
Heat maps showing the expression profiles of turnip *CDPK* genes across different tissues and developmental stages of tuberous roots. Quantitative RT-PCR was used to assess *BrrCDPK* transcript levels in total RNA samples extracted from mature plants, root, stem, leaf, and flower tissue. The developmental stages were based on transcriptional data generated by Jingjuan Li. Samples were collected on day 18 (the early stage before cortex splitting, ES), day 28 (the cortex splitting stage, CSS), and day 64 (the stage of root thickening, RTS) after sowing. The relative expression was log transformed and visualized as heat maps.

Transcriptional profiles are typically closely related to a gene function. To investigate the link between evolutionary and functional divergence of 55 *BrrCDPK* genes in turnip development and growth, the expression levels of turnip *CDPK* genes were analyzed in four different tissues (root, stem, leaf, and flower) by quantitative real-time polymerase chain reaction (qRT-PCR) and using publicly available qRT-PCR expression profiles of three root development stages^39^. A heatmap was created using Genesis 1.7.7.0 software. As shown in Fig. 3, most *BrrCDPK* genes were specifically expressed in the root, leaf, and flower, whereas only two *BrrCDPKs* (41 and 47) were highly expressed at the stem. Additionally, *BrrCDPK46* was highly expressed in all organs except the stem. The root-specific *CDPKs* can be targets for the breeding of flourishing roots and waterlogging-tolerant cultivars because of the need for tuberous root and turnip’s vulnerability to flooding.

To analyze gene expression in different stages of tuberous root development, a heat map of different root development stages was made using the transcriptional data generated by Jingjuan Li^39^. Samples were collected on day 18 (the early stage before cortex splitting, ES), day 28 (the cortex splitting stage, CSS), and day 64 (the stage of root thickening, RTS) after sowing, respectively. Additionally, every stage had two independent biological replicates. The samples were labeled as ES1, CSS1, and RTS1 for the first biological replicate, and ES2, CSS2, and RTS2 for the second replicate. As Fig. 3 shows, most *BrrCDPK* genes were expressed at the early stage before cortex splitting (ES, day 18); six genes (*BrrCDPK5*, 11, 20, 21, 22, and 25) were not found in the transcriptional data because of their low expression levels in the root. Additionally, 19 *BrrCDPKs* were expressed in both replicates of ES, three genes (*BrrCDPK35*, 36, and 39) in CSS, and six in RTS.

To observe the functional divergence between homologous genes, the expression levels of different tissues and stages were analyzed. As shown in Fig. 3, *BrrCDPK4* and *BrrCDPK36*, *BrrCDPK7* and *BrrCDPK51*, *BrrCDPK13* and *BrrCDPK54*, and *BrrCDPK34* and *BrrCDPK52* were expressed in the same tissues. However, other paralogs were differentially expressed in different tissues. For example, *BrrCDPK1* was expressed in the root and flower. Its homologous gene *BrrCDPK21* exhibited high expression in the root and leaf, similar to paralogs at different root stages. Orthologs such as *BrrCDPK8* and *BrrCDPK53*, *BrrCDPK17* and *BrrCDPK32*, and *BrrCDPK33* and *BrrCDPK48* were highly expressed at ES, and two paralogous gene pairs (*BrrCDPK35* and *BrrCDPK43* and *BrrCDPK39*, and *BrrCDPK47*) had relatively high expression at ES and CSS. However, some orthologs exhibited rather different patterns of expression. For instance, *BrrCDPK12* and *BrrCDPK55* were expressed at RTS and ES, respectively. Overall, some homologous genes were involved in development and growth of the same organs or at the same stages, whereas some showed functional divergence and were involved in different areas of development and growth.

### Responses to abiotic and biotic stresses

**Figure 4:**
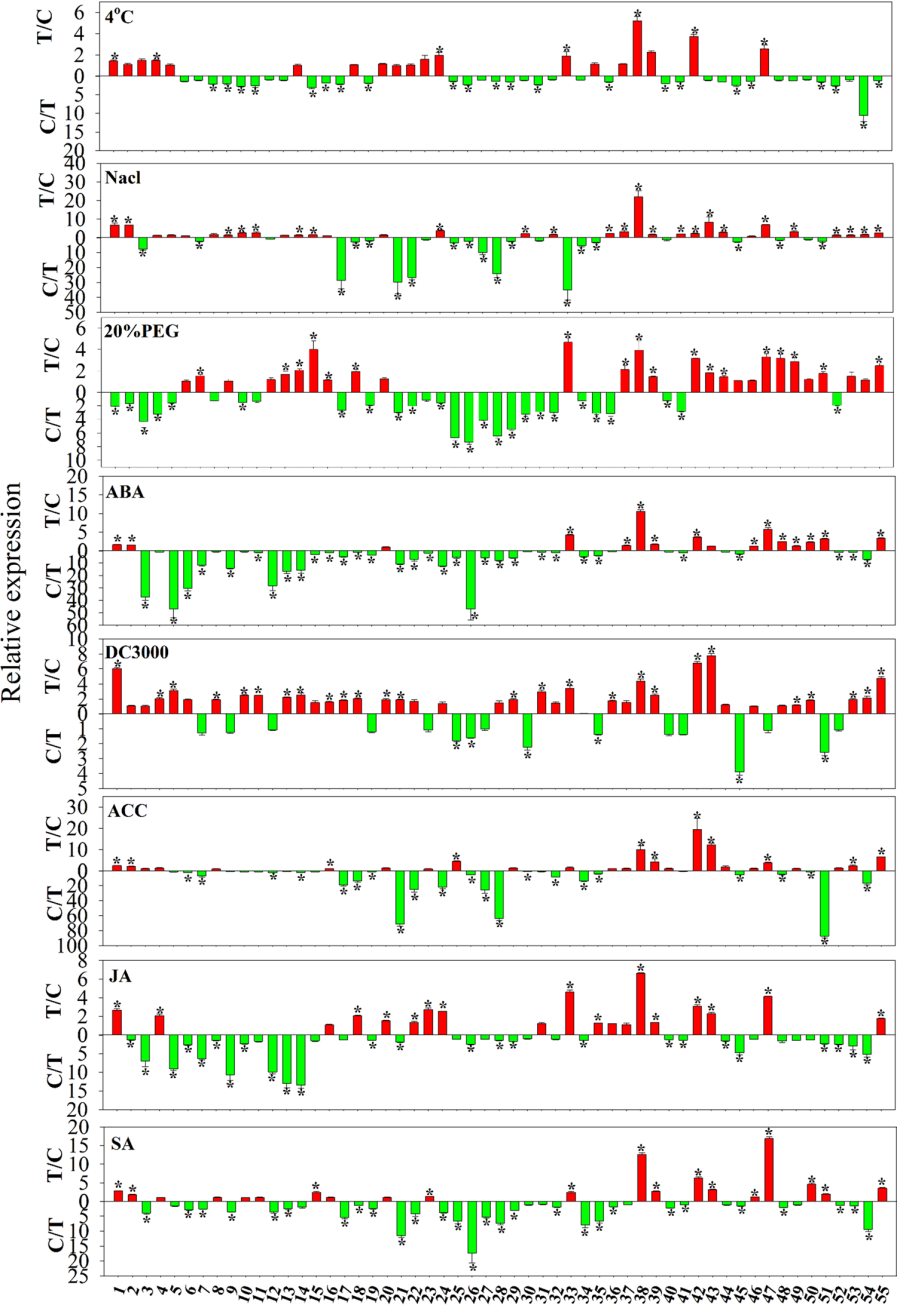
Differential expression of turnip *CDPK* genes under different stresses. Quantitative RT-PCR analyses were performed and expression values were calculated using the 2^−△△CT^ method. Data are mean values ± standard error obtained from three replicates. Red indicates upregulated genes and green downregulated genes. Asterisks denote statistically significant differences (t-test, p < 0.05).

Plants are regularly challenged by abiotic and biotic stresses. While CDPK may be involved in responding to abiotic and biotic stimuli^37,40^, information related to CDPK involvement in stress responses in turnip is limited. To gain insight into the functions of BrrCDPKs in abiotic and biotic stress responses, the expression levels of 55 *BrrCDPKs* were examined by qRT-PCR. Seedlings were harvested after being exposed to cold, salt, drought, ABA, *pst* DC3000, 1-aminocyclopropane-1-carboxylic acid (ACC; the precursor of ET), jasmonic acid (JA), and salicylic acid (SA). As shown in Fig. 4, each *BrrCDPK* gene typically responded to several treatments, with each relevant treatment altering expression of multiple *BrrCDPK* genes. For example, *BrrCDPK1* responded to seven treatments (cold, salt, ABA, *pst* DC3000, ACC, JA, and SA), and eight genes (*BrrCDPK1*, 4, 24, 33, 38, 39, 42, and 47) were induced after 1 h of cold treatment. We also calculated the number of significantly expressed (>2.5 fold) genes responding to every treatment. We found that *BrrCDPK38* and *BrrCDPK47* responded to all abiotic treatments. *BrrCDPK38* and *BrrCDPK42* were significantly expressed with *pst* DC3000, ACC, JA, and SA treatments. Interestingly, *BrrCDPK38* was upregulated with all eight treatments, suggesting that the function of BrrCDPK38 was very wide. Generally, 39 *BrrCDPK* genes responded to *pst* DC3000 treatment, which was the maximum number of upregulated genes for any treatment, suggesting that most BrrCDPKs were involved in responding to pathogens. However, the cold and JA treatments only resulted in the upregulation of 19 *BrrCDPKs*. Additionally, we analyzed the expression of homologous genes in the eight treatments. Three pairs (*BrrCDPK13* and *BrrCDPK54*, *BrrCDPK26* and *BrrCDPK27*, and *BrrCDPK38* and *BrrCDPK42*) were showed consistent expression, with both members of each gene pair upregulated or downregulated together. Among them, *BrrCDPK26* and *BrrCDPK27* were downregulated in all treatments, whereas *BrrCDPK38* and *BrrCDPK42* were upregulated under all conditions. The remaining paralogous genes showed different expression levels with at least one stress treatment; *BrrCDPK35* and *BrrCDPK43* responded to the seven treatments differently and only had similar expression patterns with JA treatment. *BrrCDPK12* and *BrrCDPK55* were expressed differently under salt, ABA, *pst* DC 3000, ACC, JA, and SA stress (Fig. 4). The remaining orthologs responded to one to five treatments differently. These results further suggested that homologous genes in turnip showed functional divergence.

### Interactions of BrrCDPKs with BrrRbohs

**Figure 5:**
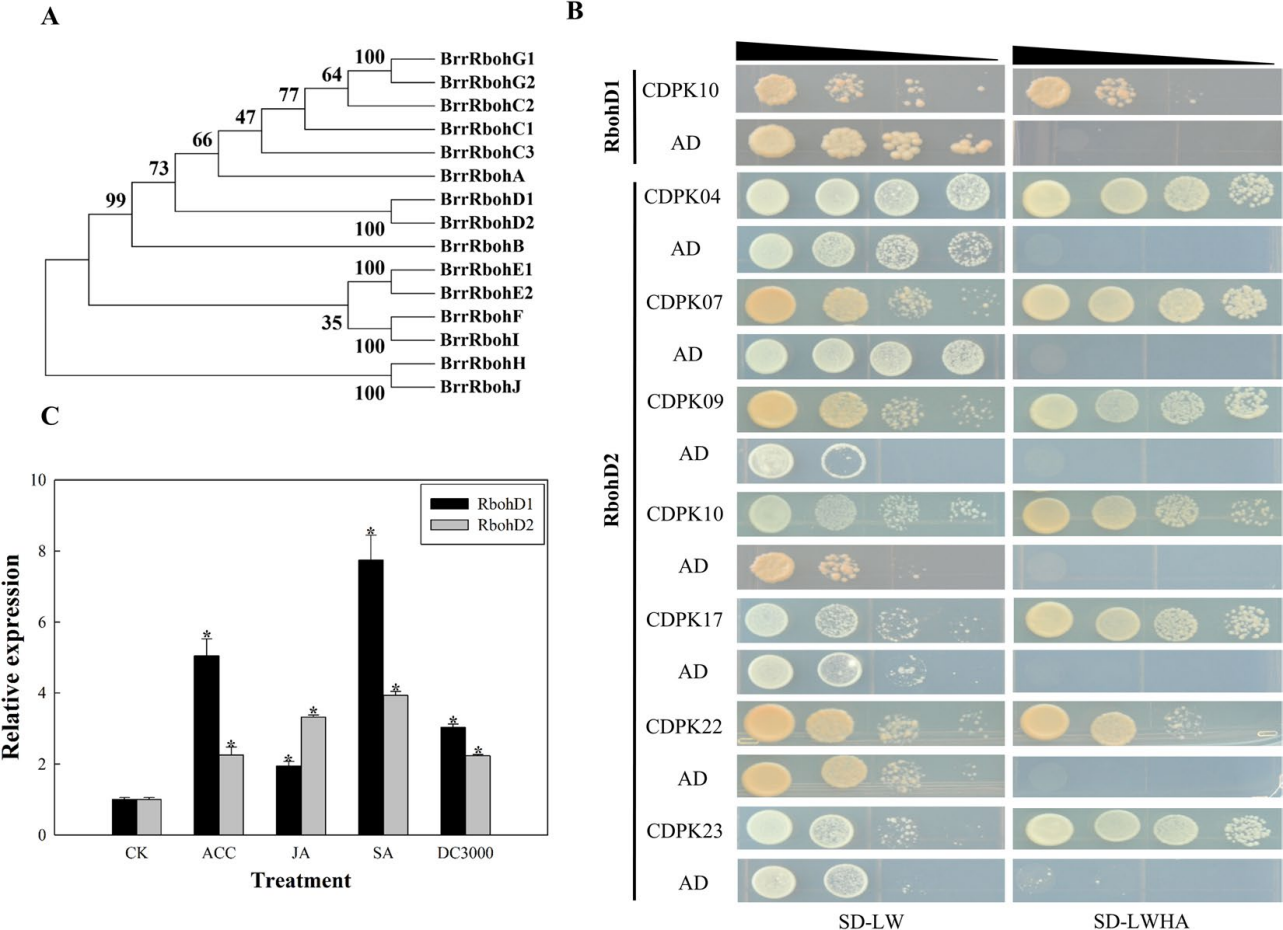
Interaction of BrrCDPKs with BrrRboh proteins. (**A**) Phylogenetic relationship of turnip Rbohs. A neighbor-joining tree was created for 15 turnip Rboh proteins using the MEGA7.0 program with 1000 bootstrap replicates. (**B**) Yeast two-hybrid analysis of interactions between CDPK and Rboh proteins in turnip. The yeast cells of strain AH109 containing the indicated plasmid combinations were grown on either the nonselective (SD-LW) or selective (SD-LWHA) media. AD is the empty pGADT7 vector. (**C**) Expression profiles of BrrRbohD1 and BrrRbohD2 under ACC, JA, SA, and *pst* DC3000 stress. Quantitative RT-PCR analyses were performed and expression values were calculated using the 2^−△△CT^ method. Data are mean values ± standard error obtained from three replicates. Red indicates upregulated genes and green indicates downregulated genes. Asterisks denote statistically significant differences (t-test, p < 0.05).

The Rboh gene family is one of the substrates of CDPK^27,30^. To date, no studies have reported the relationship of CDPKs and Rbohs in turnip. To investigate the relationship of BrrCDPKs and BrrRbohs, we selected 15 *BrrRboh* genes in turnip (Fig. 5A). Of these, we cloned seven *BrrRboh* genes, including *BrrRbohC3*, *BrrRbohD1*, *BrrRbohD2*, *BrrRbohE2*, *BrrRbohF*, *BrrRbohG1*, and *BrrRbohH*. After sequence confirmation, the correct CDPKs and Rbohs were subcloned into pGBKT7 (BD) and pGADT7 (AD) vectors, respectively. According to the results of the yeast two-hybrid assay, BrrRbohD1 interacted only with BrrCDPK10, and BrrRbohD2 interacted with BrrCDPK4/7/9/10/17/22/23 (Fig. 5B). Furthermore, Fig. 5C shows that *BrrRbohD1* and *BrrRbohD2* were upregulated with ACC, JA, SA, and DC3000 treatment, with *BrrRbohD1* upregulated by 5-, 2-, 7-, and 3-fold, respectively. *BrrRbohD2* was up-regulated 2-, 3-, 4-, and 2-fold, respectively.

### H_2_O_2_ level in guard cells and stomatal aperture of turnip treated by *pst* DC3000

**Figure 6:**
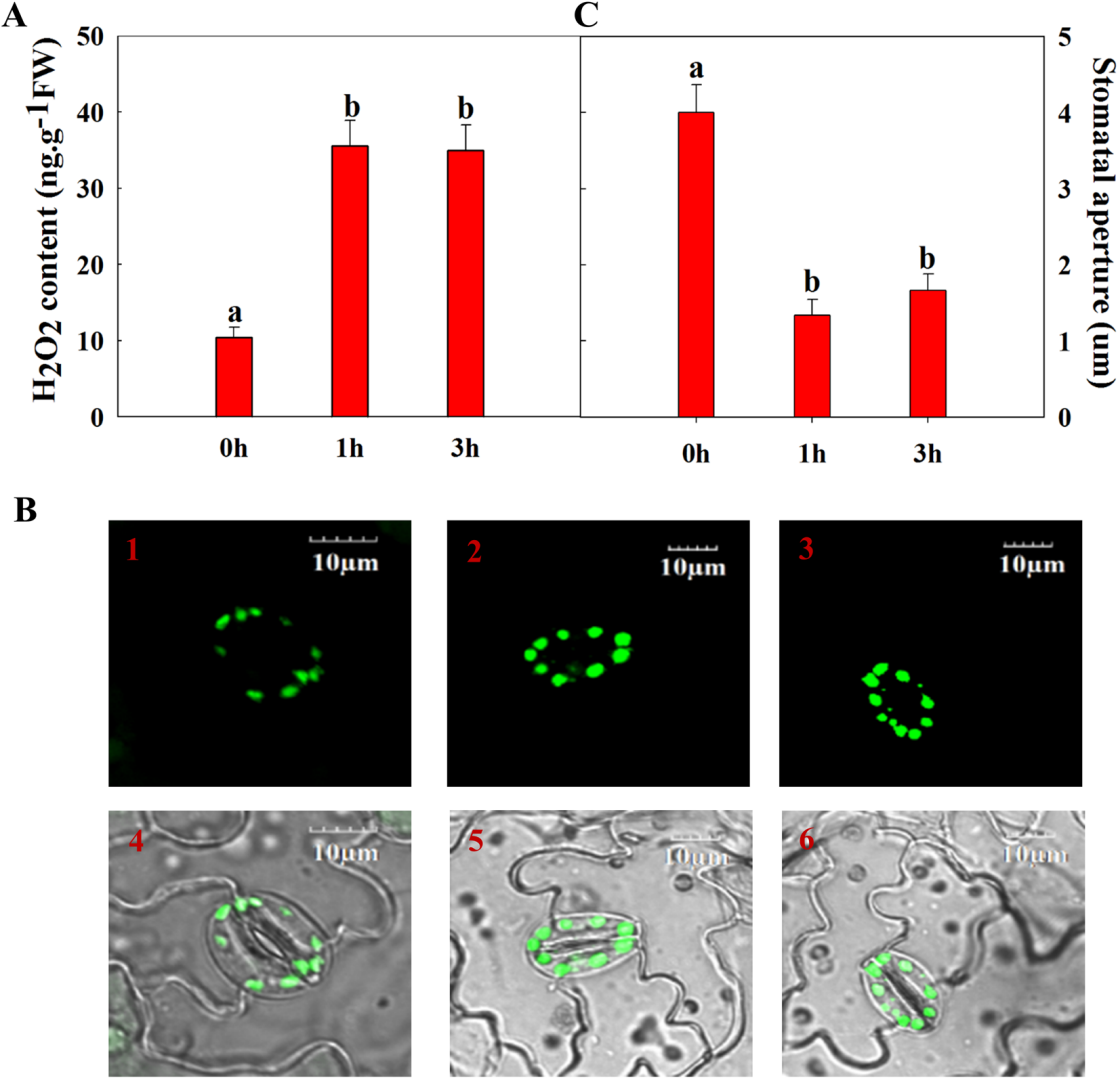
H_2_O_2_ in guard cells and stomatal closure. (**A**) H_2_O_2_ content in leaves of turnip treated with *pst* DC3000 for 0, 1, and 3 h. Data are mean values ± standard error (SE) obtained from three replicates. Different letters within a column indicate a significant difference (p < 0.05; Tukey’s test). (**B**) DC3000-induced production of H_2_O_2_ by turnip leaf guard cells. Epidermal pieces of turnip leaves without (1 and 4) or with 1 h (2 and 5) or 3 h (3 and 6) of *pst* DC3000 treatment were loaded with 50 µmol/L H2DCF-DA for 10 min. Photographs were taken using a laser-scanning confocal microscope. Pictures 1–3 are fluorescent images, 4–6 are overlapped images. (**C**) Changes in stomatal aperture of turnip leaves treated with *pst* DC3000 for 0, 1, and 3 h. Stomatal aperture size (mean values ± SE, n = 90 stomata from three leaves) of leaves treated with *pst* DC3000 for 0, 1, and 3 h. Different letters within a column indicate a significant difference (p < 0.05; Tukey’s test).

Interestingly, most CDPKs were upregulated under *pst* DC3000 treatment, and BrrRbohD1/D2 were clearly involved in *pst* DC3000 defense. Additionally, rapid generation of H_2_O_2_ during the oxidative burst has been recognized as a central component of plant defense responses to pathogens^41–43^. Moreover, a ROS burst is involved in stomatal closure associated with defense and cell death responses^44,45^. Therefore, we analyzed the H_2_O_2_ level in guard cells and stomatal apertures at 0, 1, and 3 h after treatment with *pst* DC3000. As Fig. 6A shows, the level of H_2_O_2_ at both 1 and 3 h was approximately thrice that at 0 h. We also observed the state of stomata and stomatal apertures. As shown in Fig. 6B, the stomata were opening at 0 h and almost closing at 1 and 3 h. The concentration of H_2_O_2_ was much higher at 1 and 3 h than 0 h. Additionally, stomatal aperture size at 0 h was almost thrice that at 1 and 3 h (Fig. 6C).

## Discussion

*CDPK* genes have been found in plant genomes, such as *Arabidopsis* ^11^, rice^46^, wheat^47^, cassava^48^, and grape^49^. Moreover, CDPKs play important roles in biotic and abiotic stresses. In this study, we identified 55 *CDPKs* in turnip through a genome-wide database search based on conserved domains and similarities to known CDPKs. Only 34, 31, 26, 35, and 25 *CDPKs* have been identified in *Arabidopsis*, rice, wheat, maize, and canola, respectively, much fewer than the number found here in turnip^11–15^. Similar to turnip, however, the *CDPK* gene family in soybean has been expanded to 50 members^16^. A phylogenetic tree was constructed that divided the turnip *CDPKs* into four major subfamilies that agreed with the subgroups of *Arabidopsis* ^11^. However, every subfamily in turnip had more members than that in *Arabidopsis*. Even the simple subgroup had five members, more than the three found in *Arabidopsis* ^11^. Additionally, 15 pairs of orthologous genes in turnip were located on two duplicated segments. This indicated that they were derived from segmental duplication because most paralogous genes were not physically adjacent to each other and were instead located on different chromosomes, such as *BrrCDPK12* and *BrrCDPK55*. This result indicated that segmental duplication and genome rearrangement occurred during turnip evolution^50^. This phenomenon may be the cause of the expansion of the *CDPK* gene family in turnip.

Gene divergence is a significant process in the evolution of novel functions^51^. To investigate the functional divergence of BrrCDPKs, we analyzed the expression of 21 pairs of orthologous genes. In this study, all the *Ka/Ks* values for 21 homologous genes were less than one, indicating a functional constraint with purifying selection on the genes^52^. In different tissues, only four pairs were expressed in the same tissues, whereas members of other pairs were expressed in different tissues (Fig. 3). Three pairs were expressed at the same stage of root development. Others were expressed at different stages or not expressed in the root, similar to the patterns observed in other plant species such as cucumber^53^, soybean^16^, and pepper^54^. Plant growth, development, and response to environmental stresses are regulated at the transcriptional level^55–57^. We, therefore, analyzed the expression of *BrrCDPKs* under different stress conditions. Our study provided evidence that most *BrrCDPKs* had remarkable responses to both abiotic and biotic stress, which was consistent with the results obtained in *Arabidopsis* ^11,20^. Many CDPKs were able to respond to several stresses; BrrCDPK38 responded to eight treatments. By contrast, multiple *CDPK* genes could be affected by one stress treatment. For instance, 39 genes were upregulated by *pst* DC3000 stress. Additionally, nine pairs were differentially expressed with more than four different stress treatments, whereas three pairs remained the same. Interestingly, one pair, BrrCDPK26 and BrrCDPK27, was downregulated in all treatments, whereas the BrrCDPK38 and BrrCDPK42 pair was upregulated, indicating that these two pairs showed no functional divergence. Through the analysis of these results, we found that different *BrrCDPK* genes were specifically expressed in different tissues and developmental stages and responded to different stresses, showing the functional diversity of BrrCDPKs. Additionally, some homologous genes showed functional divergence, with their responses to different stresses potentially extending novel functions^51^.

Many studies have shown that some *CDPK* genes are involved in anti-pathogen responses; Rbohs, one of the substrates of CDPK, played an important role in plant immunity^27,30,58–61^. StCDPK5 also interacted with StRbohC^27^. Studying the interactions between BrrCDPKs and BrrRbohs, we found that BrrRbohD1 only interacted with BrrCDPK10, whereas BrrRbohD2 interacted with BrrCDPK4/7/9/10/17/22/23 through the yeast two-hybrid assay. This result confirmed that BrrCDPKs interacted with BrrRbohs. Moreover, according to our data, we found that most CDPKs were upregulated under *pst* DC3000 treatment. Multiple *BrrCDPK* genes also responded to phytohormones such as ACC, JA, and SA that participate in biotic stress signaling pathways^62,63^. To identify the function of BrrRbohD1/2 in defense against *pst* DC3000, we determined that both were upregulated under ACC, JA, SA, and *pst* DC3000 stress, indicating that they are involved in resistance to pathogens and that *BrrRbohD1* was redundant with the dominant *BrrRbohD2* gene. Additionally, *BrrCDPK4/10/17* were significantly upregulated under *pst* DC3000 stress where the expression of each increased by almost 2-fold. We, therefore, hypothesized that BrrCDPK4/10/17 interacted with BrrRbohD1/D2 to facilitate resistance to *pst* DC3000.

The accumulation of H_2_O_2_ is one of the earliest events in the hypersensitive response (HR)^41^ by which pathogens are recognized. Additionally, H_2_O_2_ is rapidly produced after pathogen infection^41^. Previous studies have demonstrated that AtCDPK1 and AtRbohD produced greatly increased ROS to protect against infection with *pst* DC3000^60^. CDPKs may also function upstream of ROS production^30,64^ and may regulate RbohD to accumulate ROS. As Fig. 6A shows, the H_2_O_2_ content at 1 and 3 h was almost thrice that at 0 h suggesting that H_2_O_2_ accumulated after *pst* DC3000 inoculation. Additionally, the stomata are a natural entry site for harmful microbes, with many microbes invading plant tissues through the stomata. For example, *pst* DC3000 enters plants via open stomata on *Arabidopsis* leaves^65^. Similarly, stomatal closure can prevent disease incidence; leaf inoculation with *pst* DC3000 decreased stomatal aperture within 1 to 2 h of inoculation^65^. H_2_O_2_ functions in both MAMP- and ABA-induced stomatal closure^66^. As shown in Fig. 6B, the stomata were almost closed within 1 and 3 h of inoculation and H_2_O_2_ was found in the guard cells. Moreover, as seen in Fig. 6C, the stomatal apertures at 1 and 3 h were smaller than at 0 h. We, therefore, concluded that H_2_O_2_ can be induced by inoculation with *pst* DC3000 and that increased H_2_O_2_ can regulate stomatal closure to prevent pathogen entry. Previously, MAMP and ABA have been shown to trigger ROS production by activating Rbohs, especially RbohD^67,68^. Furthermore, CDPK signaling is known to occur upstream of H_2_O_2_^69^, and MAMP-triggered ROS production has been shown to regulate stomatal closure^21^. Consistent with these earlier results, we hypothesize that *pst* DC3000-activated BrrCDPK4/10/17 activated BrrRbohD1/D2 to produce H_2_O_2_ that induced stomatal closure to prevent *pst* DC3000 infection.

## Conclusions

At present, few studies of the *CDPK* family have been reported in turnip and the function of *CDPK* family members remain to be clarified. In our study, 55 *BrrCDPK* genes were identified that were phylogenetically clustered into four subfamilies. Chromosome locations indicated that the turnip *CDPK* gene family exhibited expansion by segmental duplication and genome rearrangement. Moreover, gene expression profiles showed that different *BrrCDPKs* were expressed in specific tissues or stages. The transcript levels of *BrrCDPKs* showed that they were involved in responses to abiotic and biotic stress and that paralogs showed functional divergence. Subsequently, our data revealed that BrrRbohD1 only interacted with BrrCDPK10 and that BrrRbohD2 interacted with BrrCDPK4/7/9/10/17/22/23. Additionally, most of these were involved in defense against *pst* DC3000. We also observed that the accumulation of H_2_O_2_ and stomatal closure were induced by inoculation with *pst* DC3000. We predicted that BrrCDPK4/10/17 regulated BrrRbohD1/D2 to increase the level of H_2_O_2_ and regulate stomatal closure to assist in disease resistance. These results may lay the foundation for further studies of turnip *CDPK* genes and provide candidate genes for the development of plant immunity.

## Methods

### Identification of the *CDPK* gene family in turnip

To identify *CDPK* genes in turnip, we obtained genes from the Turnip Genome Database in JBrowse website (http://www.bioinformatics.nl/brassica/index.html?data=bras_tp%2Fdata&loc=A01%3A11418033.17127994&tracks=DNA%2CGenes%2CTranscripts&highlight=). The protein domains and functional sites were examined using the domain analysis program ps_scan.pl^70^. Protein sequences, including protein kinase domains (PS50011) and EF-hand calcium-binding domains (PS50222), were extracted and used to search against the GenBank nonredundant (Nr) protein database. Then, we excluded the sequences that were CDPK-related proteins, calcium/calmodulin-dependent proteins, and calcium and calcium/calmodulin-dependent protein kinases. The remaining proteins were regarded as BrrCDPKs. The number of EF hands was predicted by SMART (http://smart.embl-heidelberg.de/)^71^. The molecular weight, theoretical pI, and grand average of hydropathicity were predicted by the ProtParam tool of ExPaSy (http://web.expasy.org/protparam/)^72^. Myristoylation and palmitoylation sites and N-terminal acylation were predicted using PlantsP (http://plantsp.genomics.purdue.edu/myrist.html), CSS-Palm 3.0^73^, and NetAcet 1.0^74^ software, respectively.

### Sequence alignment and phylogenetic analyses

Fifty-five full-length turnip CDPK protein sequences were aligned via the MAFFT 7.0 program and then used to construct a phylogenic tree via the neighbor-joining method of MEGA7.0 software^75^. Bootstrap values were estimated to assess the relative support for each branch with 1,000 replicates. Gene Structure Display Server (http://gsds.cbi.pku.edu.cn/) was used to analyze the gene exon/intron structure^76^.

### Genomic distribution and synteny analysis

*BrrCDPKs* were mapped on chromosomes by confirming their detailed chromosomal position supplied in the Turnip Genome Database. The segmental and tandem duplication regions were obtained using the MCscanX software^77^. For synteny analysis, synteny blocks of the turnip gene were visualized using Circis (http://circos.ca/).

### Divergence time estimation

Synonymous (*Ks*) and nonsynonymous (*Ka*) substitution rates were calculated using the codeml program of PAML4^78^. The divergence time (T) of turnip *CDPK* gene pairs was calculated using the formula T = *Ks*/2r, where r represents the divergence rate of 1.5 × 10^−8^ mutations per *Ks* site per year.

### Plant growth, and sample treatments

Seeds of turnip were germinated in sterile culture dishes with wet filter paper for 24 h in the dark at 23 °C. The seedlings were grown in perlite and Hoagland’s nutrient solution under controlled conditions (28 °C day/25 °C night cycle, 200 mmol photons m^−2^ s^−1^ light intensity, and 75−80% relative humidity). After 4 w of germination, the seedlings were subjected to different treatments. For cold treatment, plants were exposed to 4 °C. For salt and drought stress, 250 mM NaCl or 20% (w/v) polyethylene glycol was sprayed on both sides of each leaf. After 1 h, the leaves were cut and frozen in liquid nitrogen immediately, and stored at −80 °C. For JA, SA, ET, and ABA treatments, 7.5 g/L JA and 1 mM SA, ACC, or ABA was sprayed on the leaves, and sampled after 30 min^16^. Seedlings were soaked in *Pst* DC3000 (*Pseudomonas syringae* pv. Tomato DC3000) bacterium solution for 10 min and the leaves were harvested after 1 h.

### Development-related expression profiles

The roots, stems, leaves, and flowers of mature plants were sampled from the greenhouse using scissors and stored at −80 °C until use for tissue-specific expression analysis.

For the three root developmental stages, the expression levels from RNA-Seq data were obtained from previous research^39^. Samples were collected on day 18 (the early stage before cortex splitting, ES), day 28 (the cortex splitting stage, CSS), and day 64 (the stage of root thickening, RTS) after sowing. Additionally, each stage included two independent biological replicates.

### RNA extraction and Analysis of quantitative real-time PCR (qRT-PCR)

Total RNA was isolated from turnip samples using TRIzol reagent (Invitrogen, Carlsbad, CA, USA) according to the manufacturer’s instructions. RNA quality was determined using a NanoDrop ND1000 spectrophotometer (NanoDrop Technologies, Wilmington, DE, USA). Then, DNA-free total RNA (5 μg) was used for first-strand cDNA synthesis by Superscript III reverse transcriptase (Invitrogen) following the manufacturer’s instructions. The primers were designed by Primer-BLAST (http://www.ncbi.nlm.nih.gov/tools/primer-blast/index.cgi?LINK_LOC = BlastHome), offering the following parameters: 150–200 bp of PCR product size, Nr database, 57–63 °C primer melting temperatures, and *B. rapa* (*taxid:3711*) for ‘Organism’. The turnip *tublin-β* gene was used as the control (Table S1). Each sample included three biological and technical replicates. PCR reactions were performed under the following conditions: 40 cycles of 5 s at 95 °C, 15 s at 60 °C, and 34 s at 72 °C using FastStart Universal SYBR Green Master (Rox, Roche, Indianapolis, IN, USA).

### Yeast two-hybrid analysis

The full-length *BrrCDPKs* were cloned and subcloned into pGBKT7 (BD) vectors. *BrrRboh* gene sequences were also obtained from the Turnip Genome Database in JBrowse website. Then, BrrRbohs were cloned and subcloned into pGADT7 (AD) vectors. The primers are shown in Table S2. The plasmids of CDPK and RBOH were transformed into yeast strain Y2H and Y187 separately. After being plated on three kinds of media, namely, SD-leucine-tryptophan (SD-LW), SD-leucine-tryptophan-histidine (SD-LWH), and SD-leucine-tryptophan-histidine-adenine (LWHA), the yeast colonies were grown at 28 °C for 2–5 d before being photographed.

The transformants were cultivated in yeast extract peptone dextrose media and were then diluted to 1/10, 1/100, and 1/1000. Two microliters of the serially diluted yeast cells was spotted on SD-LW, SD-LWH, and SD-LWHA media and grown at 28 °C for 2–5 d before being photographed.

### Analysis of H_2_O_2_ concentrations and stomatal apertures

Seedlings were soaked in *pst* DC3000 bacterium solution for 10 min and the leaves were harvested after 1 and 3 h. Leaves were sampled before treatment as a control. The measurement of H_2_O_2_ concentrations was performed following the manufacturer’s instructions for the H_2_O_2_ assay kit.

The leaf abaxial epidermis was stained for 10 min by H_2_O_2_ probes (H2DCF-DA, 50 µmol/L)^79^, and a laser-scanning confocal microscope was used to observe the state of stomas and the concentrations of H_2_O_2_ in guard cells. The fluorescent light was excited with the 488-nm laser line and emission was captured using a 505–530 band-pass filter. Then, we calculated the stomatal aperture. The measurement of stomatal apertures was performed using an eyepiece micrometer. The size of a stomatal aperture is the maximum distance between the inside of two guard cells. For each time point, we analyzed three leaves, with 30 stomas evaluated randomly per leaf.

## Electronic supplementary material

Supplementary Tables
