# Supplementary material for: Genome-wide survey indicates diverse physiological roles of the turnip (Brassica rapa var. rapa) calcium-dependent protein kinase genes
Source: Sci Rep. 2017 Nov 17;7:15803. doi: 10.1038/s41598-017-16102-0 (PMC5693941; doi:10.1038/s41598-017-16102-0)
Supplement: Supplementary file 1 — Supplementary Tables [file 41598_2017_16102_MOESM1_ESM.pdf]

Supplementary Tables

Genome-wide survey indicates diverse physiological roles of the turnip (*Brassica rapa* var. *rapa*) calcium-dependent protein kinase genes

Qiuli Wang[1,2,3,4], Xin Yin[2,3,4,5], Qian Chen[2,3,4], Nan Xiang[2,3,4], Xudong Sun[2,3,4], Yunqiang Yang[2,3,4*], and Yongping Yang[2,3,4*]

[1]School of Life Sciences, Yunnan University, Kunming, 650091, China

[2]Key Laboratory for Plant Diversity and Biogeography of East Asia, Kunming Institute of Botany, Chinese Academy of Science, Kunming 650204, China

[3]Plant Germplasm and Genomics Center, Kunming Institute of Botany, Chinese Academy of Sciences, Kunming 650201, China

[4]Institute of Tibetan Plateau Research at Kunming, Kunming Institute of Botany, Chinese Academy of Sciences, Kunming 650201, China

[5]University of Chinese Academy of Sciences, Beijing 100049, China

*Corresponding author:

Yunqiang Yang

E-mail: yangyunqiang@mail.kib.ac.cn

Yongping Yang

E-mail: yangyp@mail.kib.ac.cn

Phone: 86-871-65223398

Table S1 Primers used for expression analyses by qRT-PCR.

Gene name	Forward primer (5'-3')	Reverse primer (5'-3')
BrrCDPK1	CACTGGGGTTGACTACGCTT	TGTTACCGTGACCAGCCAAA
BrrCDPK2	GCAAGGAGAACTCAACGGGA	TGACGTCGTCTCTGTCTTGC
BrrCDPK3	AGCTCCCCGTTGAAAGCTAC	ATCAGCCTCAGGTCCGTAGT
BrrCDPK4	CACTGTGAAGCGTGTTGGTG	CAACATCAGCTGAGCGCAAG
BrrCDPK5	GATCGTGGTGCAGCCTCATA	TCGGTTCTGGTTGGTGCATT
BrrCDPK6	GACCGGTCAGCCAAACATTG	TCGAACAACTCTCCACCAGC
BrrCDPK7	AACAACAATGGCGAACCCAC	GGGTCACACCAAACTGTCCA
BrrCDPK8	CCCCAGAGGTACTTAGGCGA	TGGGACACCACAGAGAAGGA
BrrCDPK9	TGCGAAGGTGGTGAGTTGTT	ACAAGCCTCAACGACGCTAA
BrrCDPK10	CCAGCGAGTCAAGAACCAGA	TTGCAGGCAAGCTTTTGACC
BrrCDPK11	ACGTTCTTGGTCGTGTCCTC	AGGAGCTTCCTCTTGGGGAT
BrrCDPK12	AGGAGCTTCCTCTTGGGGAT	ACGCTGCAAACAAATGGTCC
BrrCDPK13	TCTCCGGGAGTTTGGGATGA	GTTCACGCCGTTTCTTTGGG
BrrCDPK14	ATTTCAAACACGGCCAACGG	ATAACACCAGCGCTCCAGAC
BrrCDPK15	TGAAGAAGCTGCGCTCAAGA	CATCCCAAAACGGAGGGACA
BrrCDPK16	AGGAGAATCTGGTGGTTGCG	GTGCAGGCTTGTTGAAGCTC
BrrCDPK17	ACCGAGTCAGTGTTGCAGAG	TTCCCTGTGCCTTTCTCGAC
BrrCDPK18	CAAAGCGGCAAAGAGCAGTT	AGCGTACTTCTTCCCGGTTG
BrrCDPK19	CAGCTTTGCCATAAGCACGG	CAACGGCGAGTTCTCCTTCT
BrrCDPK20	CGGAACTATGGACCAGAGGC	AAAGGCGGAACACCACTGAT
BrrCDPK21	CCGCTCTAGTTCCACCAACA	AGAGTGTAGAGGTCGCGGAT
BrrCDPK22	GATCCAGTGGAGGCACGTAG	CGGTTGAGTCATCTTGGCCT
BrrCDPK23	ACGATAACGCCGTGCATTTG	AACCCCATGCTTGTGGCATA
BrrCDPK24	TGACAAGAACGGTCGCTTGA	TCCGCTGCATCCATTAGCAT
BrrCDPK25	ACCTCACAACGTGACGAGAC	ACTGCCCGTGACCTAACTTG
BrrCDPK26	CTTGCGAAAGCTCGGGATTG	AAGTGCTCGTCGTTTCCCAT
BrrCDPK27	CTGTGGAACGGCTGGATCTT	GCTTGATCTCTCGACCCGTT
BrrCDPK28	AGACCTGCAAGAAGGCGAAA	CGGCCTAGTTCTTTCCCGAG
BrrCDPK29	TGGAGTAAGAACCAACCGCC	CGCACATGTACGTTGTTCCC
BrrCDPK30	TTGGGAAAAGAGCTAGGCCG	ACAATGTTGGCTTGTCCCGA
BrrCDPK31	TCTGCACCGAGAAGTCAACC	GGGTGCTCCGAGAGATGATG
BrrCDPK32	TTCCAGCTCGGAACTTCACC	AATATCTGAGCGGCGAGTGG
BrrCDPK33	CAGCATGCGAAGAAAGCTCC	CCGCAATCACACGAAGAACC
BrrCDPK34	TTCGTTCTTGGCCGTTACGA	CCGTAAGGGATCGCTTCTCC
BrrCDPK35	CAGGGGTTGACTACGCTTGT	AACAACTCACCACCAGCACA
BrrCDPK36	CTAGCATCTCAGAGAGCGCC	GAACTTCTTCAGGCGGGACA
BrrCDPK37	ACGTTGCTCCTGAGGTTCTG	GTACAGTATGACCCCTGCGG
BrrCDPK38	TGTCAAAGACCCTCGAGCAC	GCCTCGTCGAGTGTACTAGC
BrrCDPK39	ACCAAAAGCGTCGTCTCACT	GGAGCCGTGTTTGCATTCTG
BrrCDPK40	ACATGTCCGCAAACCAAACG	TTGTCGCTGATCTGAGTCCG
BrrCDPK41	CCATTTTGGGGTGAGACCGA	GCAAGGAGAACTCAACGGGA
BrrCDPK42	TTCGAATGCACACGGGGTTA	CTTGCAACTCTCGGGGAACT

BrrCDPK43	TACGTTCTCGGCCACAAGAC	CGACCTCGGTGCAGAGATAC
BrrCDPK44	TACCGACAAAAGCGGGTCAA	TGTCTCTGCATTGTCGCTGT
BrrCDPK45	CCGGTGATGTGGACGGTAAT	AGTGTTGGAATGCTAGGCGA
BrrCDPK46	ACGTGAAGAGAGAAGTGGCG	AGCGCTATCGTCCTCACAAG
BrrCDPK47	GATCCAACAGGACGCGAGAT	CGACTTACACGCGAAGACCT
BrrCDPK48	CCCCGAAGAATCCAAACCGA	CGGTGATCTTGGTCTGGTGG
BrrCDPK49	ATCAAGACCATTGTGGCGGT	AACAGAGAGGCCAAAGTCGG
BrrCDPK50	TGTAGATGGGGACGGGACTT	TGCAAATGTTCGTCGTTGGC
BrrCDPK51	AGCCCTGAGGGAGTTTGGTA	ACAGTTCACGCCGCTTCTTA
BrrCDPK52	GGCGAGATCTACCGGAACAG	AGACGATCGAGGACTGGTGA
BrrCDPK53	AGTGAGGAAGTCATCGCTGC	TTGAATACTGCCGTGACGCT
BrrCDPK54	GCCTCCGTACCACAATCCAA	CTGAGTGCAGAGATGCGTGA
BrrCDPK55	TGGGAGCGGTTACATCACAC	ATGCGGACATCCTCAACACC
BrrTUB2	AGGCGTGTGAGTGAGCAGTT	CATCTCGTCCATTCCTTCACCTGT

Table S2 Primers used for yeast two-hybrid assay.

Gene name	Forward primer (5'-3')	Reverse primer (5'-3')
BrrCDPK1	ATGGCCATGGAGGCCGAATTCATGGGCAATTCATGCCGCC	CCGCTGCAGGTCGACGGATCCGGCGTCTCTCATGCTAATG
BrrCDPK2	ATGGCCATGGAGGCCGAATTCATGGGTTGCTTCAGCAGCA	CCGCTGCAGGTCGACGGATCCATCAGTCAATGGAATGGAA
BrrCDPK3	ATGGCCATGGAGGCCGAATTCATGGGACACAGACACAGCA	CCGCTGCAGGTCGACGGATCCCATATTGACTCTGCGTCGG
BrrCDPK4	ATGGCCATGGAGGCCGAATTCATGGCTAACAAATCAAGAA	CCGCTGCAGGTCGACGGATCCGGAAGAGGCTACTTTCCAA
BrrCDPK5	ATGGCCATGGAGGCCGAATTCATGGCATGTCTCTGCATCA	CCGCTGCAGGTCGACGGATCCAATACTTGACAATCCTTCA
BrrCDPK6	ATGGCCATGGAGGCCGAATTCATGGAAGATGTAAAATCT	CCGCTGCAGGTCGACGGATCCCTCGACTTTGATGAAGTAA
BrrCDPK7	ATGGCCATGGAGGCCGAATTCATGGGAAATTGTTGCGGAC	CCGCTGCAGGTCGACGGATCCTTTAAAAGACATTTCACGC
BrrCDPK8	ATGGCCATGGAGGCCGAATTCATGGGAAACTGTTGTGCAA	CCGCTGCAGGTCGACGGATCCTGTCTCGCCTTCTAATTGC
BrrCDPK9	ATGGCCATGGAGGCCGAATTCATGGCCAGCGAGTCAAGAA	CCGCTGCAGGTCGACGGATCCTTCAGGAAGAAAATTCTCA
BrrCDPK10	ATGGCCATGGAGGCCGAATTCATGCATCACTTGTCTGAAA	CCGCTGCAGGTCGACGGATCCGTTTTTATTGAAAACATAA
BrrCDPK11	ATGGCCATGGAGGCCGAATTCATGGCCAGCGAATCAAGAA	CCGCTGCAGGTCGACGGATCCTTCCTTAGGCTCATGA
BrrCDPK12	ATGGCCATGGAGGCCGAATTCATGGGTAACACTTGTGTTG	CCGCTGCAGGTCGACGGATCCGAGTTTAAGAGCAATATTG
BrrCDPK13	ATGGCCATGGAGGCCGAATTCATGGGGAATTGTTGCTCCG	CCGCTGCAGGTCGACGGATCCTTTAAATGAAAGTTCACGC
BrrCDPK14	ATGGCCATGGAGGCCGAATTCATGGGAAGCTGTGTTTCAT	CCGCTGCAGGTCGACGGATCCGTATCTTAGACCTGAGGGT
BrrCDPK15	ATGGCCATGGAGGCCGAATTCATGGGAAACACATGCACGG	CCGCTGCAGGTCGACGGATCCACTCACCCTTAAACCCATT
BrrCDPK16	ATGGCCATGGAGGCCGAATTCATGGAGAAACCAAGCTCTA	CCGCTGCAGGTCGACGGATCCGTTTGGTTTGTCATCAGTT
BrrCDPK17	ATGGCCATGGAGGCCGAATTCATGGCCGGCGTAAACCTTC	CCGCTGCAGGTCGACGGATCCTAGCATCTCTTTGAAACAT
BrrCDPK18	ATGGCCATGGAGGCCGAATTCATGGGAAATTGCTTTGCCA	CCGCTGCAGGTCGACGGATCCGAAGAGCCGCGGTGGTTGT
BrrCDPK19	ATGGCCATGGAGGCCGAATTCATGGGGAACTGCTGCAGAT	CCGCTGCAGGTCGACGGATCCTTCATTGCCTAGGTTCAAT
BrrCDPK20	ATGGCCATGGAGGCCGAATTCATGGGACACAGACACAGCA	CCGCTGCAGGTCGACGGATCCCATGTTGACTCTGCGTCGG
BrrCDPK21	ATGGCCATGGAGGCCGAATTCATGGGCAATTCATGCCGTG	CCGCTGCAGGTCGACGGATCCAGCGTCTCTCATGCTAATG
BrrCDPK22	ATGGCCATGGAGGCCGAATTCATGGGTCTCTGTTTCTCTT	CCGCTGCAGGTCGACGGATCCCATTTTTCGAGAAAGCTGG
BrrCDPK23	ATGGCCATGGAGGCCGAATTCATGGGGAACTGTTGCAGAT	CCGCTGCAGGTCGACGGATCCTTCGTTGCCTAGGTTCAAA
BrrCDPK24	ATGGCCATGGAGGCCGAATTCATGGGTGGAGGAATGGAGA	CCGCTGCAGGTCGACGGATCCGTTTCTTAGACCTGAGGGC
BrrCDPK25	ATGGCCATGGAGGCCGAATTC ATGGGGAATGTATGTATTC	CCGCTGCAGGTCGACGGATCCCACTTTTGTTGATGAA
BrrCDPK26	ATGGCCATGGAGGCCGAATTCATGGGGAACTGCTGTGGAA	CCGCTGCAGGTCGACGGATCCTTTTATATCTCCATGAGAC

BrrCDPK27	ATGGCCATGGAGGCCGAATTCATGGGGAATTGCTGTGGAA	CCGCTGCAGGTCGACGGATCCCTCTGCATCACAATTATTA
BrrCDPK28	ATGGCCATGGAGGCCGAATTCATGGGAAGCTGTGTTTCGT	CCGCTGCAGGTCGACGGATCCGTTTCTTAGACCTGATGGT
BrrCDPK29	ATGGCCATGGAGGCCGAATTCATGGGTTGCTTCTGGAGTA	CCGCTGCAGGTCGACGGATCCATTTATCCTAACACGTCTC
BrrCDPK30	ATGGCCATGGAGGCCGAATTCATGGGTTGCCTCAGCAGTA	CCGCTGCAGGTCGACGGATCCATGGAAGGGAAGAAGCTTC
BrrCDPK31	ATGGCCATGGAGGCCGAATTCATGGAGAAAGCAAATCCTA	CCGCTGCAGGTCGACGGATCCATCATCAGATTTTTGAGCG
BrrCDPK32	ATGGCCATGGAGGCCGAATTCATGGCCGGCGTTAATCTAC	CCGCTGCAGGTCGACGGATCCGTGTTTCAGAGATATGCTA
BrrCDPK33	ATGGCCATGGAGGCCGAATTCATGGGTAACTGTAACGTCT	CCGCTGCAGGTCGACGGATCCAACAGGAACAGATTGTCCA
BrrCDPK34	ATGGCCATGGAGGCCGAATTCATGGGTCTCTGTTTCTCCT	CCGCTGCAGGTCGACGGATCCGACTTTCCGAGAAACAAGA
BrrCDPK35	ATGGCCATGGAGGCCGAATTCATGGGCAATTCATGTCGTG	CCGCTGCAGGTCGACGGATCCCACATCTCTCATGCTGATG
BrrCDPK36	ATGGCCATGGAGGCCGAATTCATGGAGGAGTTTGTGGACC	CCGCTGCAGGTCGACGGATCCGCCATTGATTGACTCATCA
BrrCDPK37	ATGGCCATGGAGGCCGAATTCATGGCGAGGGTTCCTTGTG	CCGCTGCAGGTCGACGGATCCAGCATTTTTCCTGAGGCTC
BrrCDPK38	ATGGCCATGGAGGCCGAATTCATGGGTGTGTGTTTCTCCG	CCGCTGCAGGTCGACGGATCCCCGAGGATTCCTGTGGCCT
BrrCDPK39	ATGGCCATGGAGGCCGAATTCATGGGAAACTGTTGCGGAA	CCGCTGCAGGTCGACGGATCCTCTTGTATCGCCATTGGCA
BrrCDPK40	ATGGCCATGGAGGCCGAATTCATGGGTAACTGCATCGCCT	CCGCTGCAGGTCGACGGATCCAACAGCAACAGATTGACCA
BrrCDPK41	ATGGCCATGGAGGCCGAATTCATGGGTTTCTGCTTCTCCA	CCGCTGCAGGTCGACGGATCCTCTGATCAGCTTCGCATCT
BrrCDPK42	ATGGCCATGGAGGCCGAATTCATGGGTGTGTGTTTCTCGG	CCGCTGCAGGTCGACGGATCCCCGAGGAATCCTGTGGCCT
BrrCDPK43	ATGGCCATGGAGGCCGAATTCATGGGCAATTCATGCCGTG	CCGCTGCAGGTCGACGGATCCAGCATCTCTCATGCTGATG
BrrCDPK44	ATGGCCATGGAGGCCGAATTCATGGGTTGTTGCGGCAGTA	CCGCTGCAGGTCGACGGATCCGTGATGAATAAGTTGCCCC
BrrCDPK45	ATGGCCATGGAGGCCGAATTCATGGCTAGTTCTCAGAGGC	CCGCTGCAGGTCGACGGATCCACTTACATTATTAGCATCG
BrrCDPK46	ATGGCCATGGAGGCCGAATTCATGGGGAACTGTTGCAGAT	CCGCTGCAGGTCGACGGATCCTTCGTTGCCTAGGTTCAAA
BrrCDPK47	ATGGCCATGGAGGCCGAATTCATGGGTAATTGTTGCGGGT	CCGCTGCAGGTCGACGGATCCTATTGCCTCGCTATTGACA
BrrCDPK48	ATGGCCATGGAGGCCGAATTCATGGGTAACTGTAACGTCT	CCGCTGCAGGTCGACGGATCCAACAGGGACAGATTGTCCA
BrrCDPK49	ATGGCCATGGAGGCCGAATTCATGTCAGACTCTCAAACTA	CCGCTGCAGGTCGACGGATCCTTCATTTGACTCTTGAGGA
BrrCDPK50	ATGGCCATGGAGGCCGAATTCATGTACGATCTTGGCCGTG	CCGCTGCAGGTCGACGGATCCGGTCTCGCCTTCTAATTGC
BrrCDPK51	ATGGCCATGGAGGCCGAATTCATGGGCAATTGTTGCGCTC	CCGCTGCAGGTCGACGGATCCAGTTGTGTCAAAAGACAGT
BrrCDPK52	ATGGCCATGGAGGCCGAATTCATGGGTCTCTGTTTCTCCT	CCGCTGCAGGTCGACGGATCCAGCAAGCGAAGAGCAGTCC
BrrCDPK53	ATGGCCATGGAGGCCGAATTCATGGGGAACTGTTGCGGCA	CCGCTGCAGGTCGACGGATCCAGCCTCACCTGCTAGTTGC
BrrCDPK54	ATGGCCATGGAGGCCGAATTCATGGGAAACTGCTGCTCTA	CCGCTGCAGGTCGACGGATCCTTTAAATGAGAGGTCACGC
BrrCDPK55	ATGGCCATGGAGGCCGAATTCATGGGTAATACTTGTGTTG	CCGCTGCAGGTCGACGGATCCGAGTTTAAGAGCAATACTA
BrrRbohC3	GCCATGGAGGCCAGTGAATTCATGGAGAGACCAGTGAGCT	CAGCTCGAGCTCGATGGATCCTGTCTTCTTTGTAGATGAT
BrrRbohD1	GCCATGGAGGCCAGTGAATTCATGAGACGAGGCAATTCAG	CAGCTCGAGCTCGATGGATCCGAAGTTCTCTTTGTGGAAG
BrrRbohD2	GCCATGGAGGCCAGTGAATTCATGAGACGAGGCAGTTCAG	CAGCTCGAGCTCGATGGATCCAAAGTTCTCTTTGTGGAAG
BrrRbohE2	GCCATGGAGGCCAGTGAATTCATGAAGTTATCTCCCAAGA	CAGCTCGAGCTCGATGGATCCGAAATTTTCCTTATGGAAC
BrrRbohG	GCCATGGAGGCCAGTGAATTCATGACTTCTGACACAGAAG	CAGCTCGAGCTCGATGGATCCCAAGTTCTCATTGTGGAAG
BrrRbohH	GCCATGGAGGCCAGTGAATTCATGAAAAATAACAGTCCTA	CAGCTCGAGCTCGATGGATCCAAAGTTTTCCTTATGGAAA
